# Ureteral Haemangiosarcoma in a Dog: Rare Primary Tumour With Unusual Metastasis to the Contralateral Kidney

**DOI:** 10.1155/crve/3429069

**Published:** 2025-02-05

**Authors:** Kacper Żebrowski, Małgorzata Kandefer-Gola, Jolanta Bujok, Wojciech Borawski, Stanisław Dzimira

**Affiliations:** ^1^Department of Pathology, Division of Pathomorphology and Veterinary Forensics, Faculty of Veterinary Medicine, Wroclaw University of Environmental and Life Sciences, Wroclaw, Poland; ^2^Department of Biostructure and Animal Physiology, Faculty of Veterinary Medicine, Wroclaw University of Environmental and Life Sciences, Wroclaw, Poland; ^3^Department and Clinic of Surgery, Faculty of Veterinary Medicine, Wroclaw University of Environmental and Life Sciences, Wroclaw, Poland

**Keywords:** dog, haemangiosarcoma, kidney, metastasis, neoplasm, ureter

## Abstract

A dog (neutered male, 11 years old, Labrador retriever) underwent abdominal ultrasound, which revealed a 7 cm diameter tumour (caudal region of the left kidney). The animal showed symptoms of weight loss, apathy, haematuria, and abdominal pain. A computed tomography (CT) scan confirmed the presence of a tumour originating from the ureter. Following surgery to remove the ureter with the attached kidney, a histopathological examination was performed. The tumour was classified as a haemangiosarcoma. After the initial recovery, 2 months after surgery, the dog was diagnosed with a tumour in the other kidney. A fine needle biopsy was carried out. A haemangiosarcoma metastasis was suspected. Neoplasms of the ureter are a rare pathology. This is the first case in which metastasis to the second kidney has been confirmed.

## 1. Introduction

Primary ureteral neoplasia in companion animals is a rare pathology [1]. Only a few cases have been reported. These include tumours of both epithelial and mesenchymal origin [2–6]. In animals, benign tumours are most commonly located in the proximal part of the ureter, whereas malignant neoplasms are located in the distal part [7, 8]. Haemangiosarcomas can also develop in the ureter [9, 10]. The case presented here is one of the rare cases of ureteral haemangiosarcoma in dogs.

## 2. Case Presentation

**Figure 1:**
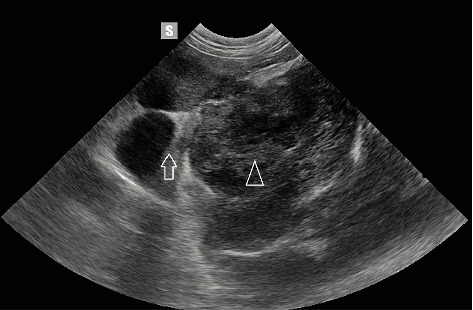
Ultrasound image of the tumour and the kidney. The study shows an extensive heteroechoic tumour (arrowhead) originating from the initial part of the ureter and completely occupying its lumen, causing obstruction and urinary stasis in the kidney (arrow).

An 11-year-old neutered male Labrador retriever was presented to the clinic with loss of appetite and occasional vomiting. The dog had recently become apathetic and had dark red urine. Clinical examination confirmed dehydration of approximately 5%. Auscultation revealed no pulmonary or cardiac abnormalities. The dog's temperature was 38.5°C. On palpation of the abdominal cavity, the animal was in severe pain, and there was a lot of tension in the abdominal wall. Blood morphology showed an elevated white blood cell count of −19.31 g/L (reference range: 5.05–16.76 g/L). Renal parameters were unchanged (Table 1). An ultrasound examination revealed a tumorous, echogenic, heterogeneous lesion with a diameter of 7 cm in the region of the left kidney (Figure 1). Because of the suspicion of a neoplasm, the animal was sent for abdominal computed tomography (CT).

CT of the abdominal cavity was performed using a 64-slice, 128-slice Simensgo.TOP CT scanner. The scan was performed on the long axis of the animal, in the dorsal abdominal position, first in the cranial direction and then in the caudal direction. The scan was performed in the precontrast phase and also after intravenous injection of the contrast agent iomeprol (Iomeron 350 mg iodine/mL, Bracco, Konstanz, Germany) at a dose of 700 mg/kg.

**Figure 2:**
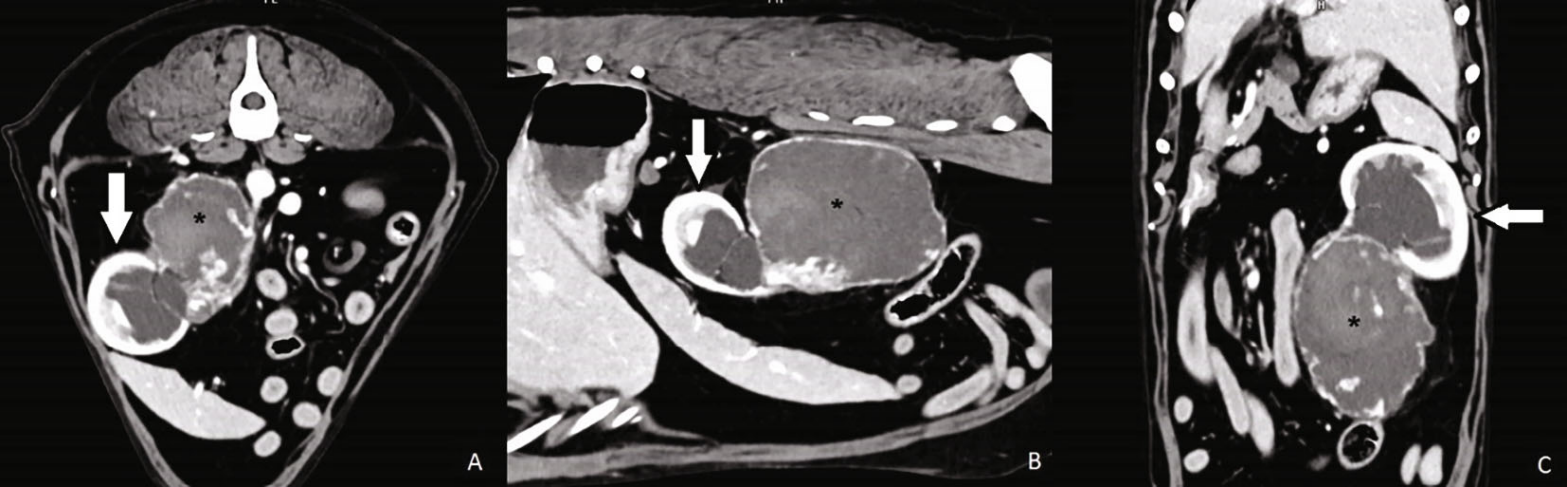
Cross-sectional multiplanar computed tomography reconstruction image with intravenous contrast injection. (A) Axial, (B) sagittal, and (C) dorsal sections show a kidney (arrow) with features of moderate hydronephrosis and a severely dilated initial portion of the ureter (⁣^∗^). The dilated part of the ureter shows irregular postcontrast enhancement both within the wall and its lumen.

The CT scan showed the presence of a proliferative lesion in the retroperitoneal space, caudal-medial to the left kidney, originating from its hilum—most likely from the left ureter. The lesion measured approximately 8.1 cm (height) × 9.6 cm (length) × 8.5 cm (width) and showed slightly heterogeneous contrast enhancement—most prominent in the peripheral part (Figure 2).

The results of the CT scan led to the decision to perform a nephrectomy. The surgery was performed 1 month after the initial diagnosis. Until surgery, the patient was treated for pain with tramadol (Tramvetol, Virbac, Carros, France) at a dose of 2 mg/kg orally every 8 h.

Prior to surgery, a complete blood count and biochemical tests were performed to assess the patient's general condition. The blood count showed regenerative anaemia and persistent inflammation (monocytosis). Renal parameters were within normal limits (Table 1). A chest x-ray excluded metastases.

**Figure 3:**
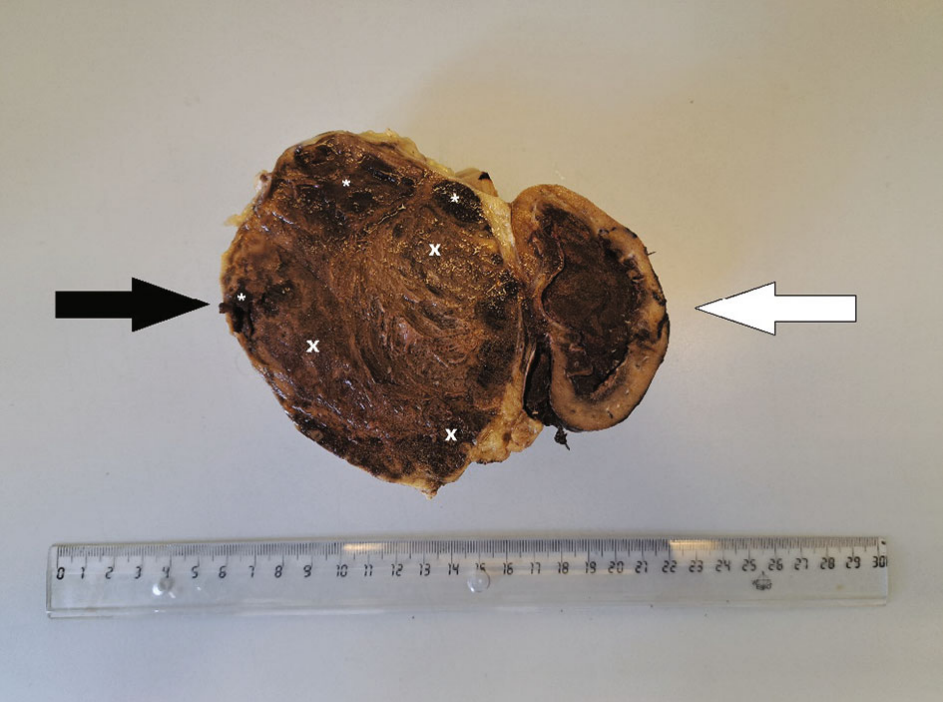
Gross view of a ureteral tumour (black arrow) with the left kidney (white arrow). There is a large area of necrosis (x) and haemorrhage (⁣^∗^) on the cross-section of the tumour.

During surgery, the tumour was removed along with the ureter from which it originated. The left kidney was also removed (Figure 3). After surgery, the patient underwent antibiotic therapy with enrofloxacin (Enroxil Flavour, Virbac, Carros, France) at a dose of 5 mg/kg for 14 days, analgesia with tramadol (Tramvetol, Virbac, Carros, France) at a dose of 2 mg/kg every 8 h for 7 days, and hydration with multielectrolyte fluids via intravenous infusion at a dose of 100 mL/h for 5 days. The recommended postoperative diet was a prepared food of pulpy consistency, light, high energy, and high protein concentration.

**Figure 4:**
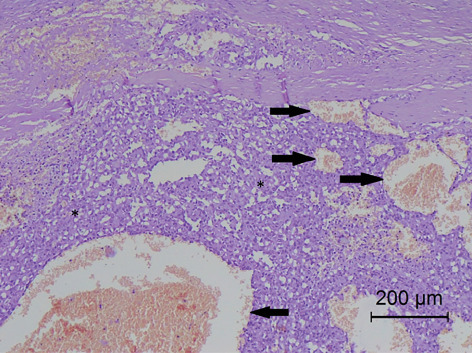
Histopathological examination revealed a ureteral haemangiosarcoma. It consists of large spindle-shaped cells, less often oval to round (⁣^∗^). Irregular blood vessels filled with erythrocytes are also present (arrows). H&E stain, ×40.

Histopathological examination revealed a proliferation of large, thin- or thick-walled, irregularly shaped blood vessels filled with erythrocytes, as well as some foci of more solid tissue. These structures were formed by large spindle-shaped cells, less frequently oval to round in shape (Figure 4). Thirty-five mitotic figures were found in 10 high-power fields (HPFs) (×40 objective). The diagnosis was ureteral haemangiosarcoma.

After surgery, the animal's appetite improved, and it urinated and defecated normally. Control blood morphology and biochemistry tests were performed 24 h after surgery (Table 1).

On the 7th day after the procedure, the animal began to vomit, became apathetic, and passed a small amount of dark urine. The complete blood count showed regenerative anaemia and inflammation (leucocytosis with monocytosis). Biochemical blood tests showed a significant increase in the activity of renal parameters (Table 1). Despite fluid therapy, renal parameters continued to deteriorate after 24 h (Table 1). Ultrasound examination showed an empty bladder and no changes in other organs. To intensify diuresis, furosemide (furosemide 5% INJ., Alfasan, Giżycko, Poland) was administered intravenously three times at a dose of 5 mg/kg, 30 min apart, and intensive fluid therapy was started. Two hours after the last administration, the bladder was still empty on ultrasound. The owners decided to start haemodialysis.

During haemodialysis, the ultrafiltration target was set at 1200 mL. 14.5 L of the patient's blood was processed within 5 h of treatment, resulting in a urea reduction of approximately 50%. The patient's diuresis returned in the third hour of haemodialysis, and the patient became polyuric after haemodialysis. During the following days, a decrease in renal parameters was observed, and renal replacement therapy was discontinued.

Two months later, an abdominal ultrasound examination revealed a 2 × 3 cm lesion in the cortex of the right kidney. Material was taken from the lesion for cytological examination by fine needle aspiration biopsy. The biopsy was performed under ultrasound guidance. The sample was composed of often large, polymorphic cells with a tendency to accumulate in larger clusters. The nuclei were medium or large, round or oval, most often with coarse chromatin. Strong anisokaryosis and anisocytosis, rather strong cell pleomorphism, and 0–2 mitotic figures were reported in HPF. A diagnosis of haemangiosarcoma was made.

The animal's clinical symptoms worsened, with more pronounced weakness and apathy. The owners decided to euthanise the animal for its well-being. The owners did not agree to a necropsy.

## 3. Discussion

Neoplasms of the ureter in dogs are rare [1]. The following cases have been described: transitional cell carcinoma [6, 11], leiomyoma [2], leiomyosarcoma [5], fibroepithelial polyps [3, 7, 12], mast cell tumour [13], spindle cell sarcoma [4], and two cases of primary haemangiosarcoma within the ureter [9, 10]. The malignant tumours are much more likely to be located in the distal part, and the benign tumours are in the proximal part of the ureter [7, 8]. In this case, the opposite was true.

The symptoms most commonly observed in animals with ureteral neoplasms are related to urinary system dysfunction: apathy, weight loss, haematuria, and severe pain on palpation of the abdominal cavity. Polydipsia and polyuria may also be seen [7]. In addition, hydroureter may develop due to the mass of the tumour and its pressure on the ureter, leading to hydronephrosis [14, 15]. In our case, the dog suffered from weight loss, severe abdominal pain, haematuria, and hydronephrosis.

Diagnosis of ureteral neoplasia in animals is usually difficult. Animals present with clinical signs at a very late stage of tumour development. Abdominal ultrasound and CT with contrast should be the gold standard to assess the possibility of surgical resection of the mass. In addition, a chest x-ray should be performed to exclude/confirm the presence of metastatic changes [9]. In the described case, no metastatic lesions in the lungs were detected on the control chest x-ray in three projections.

The recommended treatment for ureteral tumours is unilateral nephrourectomy, that is, removal of the ureter and the associated kidney. An alternative is partial ureterectomy with ureteroneocystomy, that is, reimplantation of the ureter into the bladder [16].

The prognosis for a patient with a malignant ureteral tumour is poor. These types of lesions often metastasise to organs such as the lungs or kidneys. In the case described here, the chest x-ray, abdominal ultrasound, and abdominal CT scan prior to nephrectomy showed no evidence of metastasis to other organs. Unfortunately, 1 month after the operation, a lesion was found in the cortex of the second kidney, and the cytological examination revealed a haemangiosarcoma.

The initial symptoms of haemangiosarcoma are nonspecific and usually unnoticeable to pet owners. It is only when the disease is well advanced that symptoms become more specific, for example, haematuria, anuria, and severe abdominal pain. An ultrasound or CT scan of the abdominal cavity may suggest a hyperplastic lesion within the ureter. The final diagnosis is made after a histopathological examination of the removed mass. The prognosis for patients with this type of tumour is poor.

## Figures and Tables

**Table 1:** Renal parameters in blood tests 1 month before the surgery, on the day of the surgery, 24 h after the surgery, 7 days after the surgery, 8 days after the surgery, immediately before haemodialysis, 24 h after haemodialysis, and 48 h after haemodialysis.

	**BUN**	**CREA**
Unit	mg/dL	mg/dL
Reference range	7–27	0.5–1.8
One month before the surgery	28	1.5
On the day of the surgery	15	1.6
24 h after the surgery	16	1.5
7 days after the surgery	48	9
8 days after the surgery	67	13.6
Before HD	131	16.8
24 h after HD	65	4.8
48 h after HD	34	2.7

Abbreviations: BUN = blood urea nitrogen, CREA = creatine, HD = haemodialysis.

## Data Availability

All data underlying the results are available as part of the article, and no additional source data is required.
